# Subtle Microbiome Manipulation Using Probiotics Reduces Antibiotic-Associated Mortality in Fish

**DOI:** 10.1128/mSystems.00133-17

**Published:** 2017-11-07

**Authors:** Victor Schmidt, Marta Gomez-Chiarri, Chelsea Roy, Katherine Smith, Linda Amaral-Zettler

**Affiliations:** a Ecology and Evolutionary Biology, Brown University, Providence, Rhode Island, USA; b Marine Biological Laboratory, Josephine Bay Paul Center for Comparative Molecular Biology and Evolution, Woods Hole, Massachusetts, USA; c University of Rhode Island, Fisheries, Animal, and Veterinary Science, Kingston, Rhode Island, USA; d Department of Earth, Environmental and Planetary Sciences, Brown University, Providence, Rhode Island, USA; e Department of Marine Microbiology and Biogeochemistry, NIOZ Royal Netherlands Institute for Sea Research and Utrecht University, Den Burg, the Netherlands; University of California, San Diego

**Keywords:** antibiotics, aquaculture, *Bacillus*, colonization resistance, microbial ecology, microbiome, *Phaeobacter*, probiotics, *Vibrio*

## Abstract

Prophylactic antibiotics in the aquaculture and ornamental fish industry are intended to prevent the negative impacts of disease outbreaks. Research in mice and humans suggests that antibiotics may disturb microbiome communities and decrease microbiome-mediated disease resistance, also known as “colonization resistance.” If antibiotics impact fish as they do mice and humans, prophylactic administrations on aquaculture farms may increase downstream disease susceptibility in target hosts, despite short-term pathogen control benefits. We tested the effects of antibiotics on mortality after a pathogen challenge in the *Poecilia sphenops* black molly and subsequently tested if probiotic inoculations could reverse any antibiotic-induced losses of disease resistance. We found that antibiotic treatment significantly increased fish mortality. We further found that our two candidate probiotic bacterial species, *Phaeobacter inhibens* S4Sm and *Bacillus pumilus* RI06-95Sm, were able to colonize black molly microbiomes and reverse the negative impacts of antibiotics. Despite the positive impact on survival, probiotic treatment did not influence overall microbiome community structure or diversity. Our results suggest that subtle manipulations of microbiome composition can have dramatic impacts on host phenotype. The results of this study have implications for how antibiotic-treated microbiomes can be restored and suggest that small-scale additions may be as effective as wholesale transplants.

**IMPORTANCE** Prophylactic antibiotics are widespread in the aquaculture industry and are used where vaccination is impossible or overly expensive. If antibiotics impact fish as they do mice and humans, prophylactic administrations in aquaculture and ornamental fish farms may increase downstream disease susceptibility in target hosts, despite short-term pathogen control benefits. Recent research has suggested that their use exacerbates bacterial outbreaks by creating sterile, nutrient-rich environments for invading pathogens to colonize and could help to explain rising economic costs of bacterial outbreaks in aquaculture. Our findings suggest a long-term cost of prophylactic antibiotic use and demonstrate a probiotic-based solution that does not rely on full microbiome community transplantation.

## Abstract (precis)

Prophylactic antibiotics are widespread in the aquaculture industry and are used where vaccination is impossible or overly expensive. If antibiotics impact fish as they do mice and humans, prophylactic administrations in aquaculture and ornamental fish farms may increase downstream disease susceptibility in target hosts, despite short-term pathogen control benefits. Recent research has suggested that their use exacerbates bacterial outbreaks by creating sterile, nutrient-rich environments for invading pathogens to colonize and could help to explain rising economic costs of bacterial outbreaks in aquaculture. Our findings suggest a long-term cost of prophylactic antibiotic use and demonstrate a probiotic-based solution that does not rely on full microbiome community transplantation.

## INTRODUCTION

Microbiomes represent a diverse ecosystem of host-associated microbes and play an important role in host health, development, and nutrition (1). In fish, the microbiome may protect its host from colonization by and proliferation of the highly diverse pathogens found in the aquatic environment (2). This protection, termed "colonization resistance," results from resident microbial competition for resources or niche space, or from direct inhibition of invading pathogens via competitive interactions (3–5).

Colonization resistance has been best studied in mice and humans, particularly in the context of antibiotic treatment. Antibiotic treatment disturbs mammalian microbiome diversity and reduces the associated colonization resistance, often resulting in an increased risk of pathogen infection (6–8). For example, *Clostridium difficile* infects human gastrointestinal tracts, causing severe diarrhea, weight loss, and death. Treatment requires cycles of antibiotics that often lead to recurrent drug-resistant infections and drastically reduced fecal microbiome diversity (9, 10). Interestingly, fecal inoculations with a microbiome from a healthy individual into the intestine of a *C. difficile-*infected patient can bring community stability to the infected intestine, restore colonization resistance, and prevent subsequent infection (11). These results generated the hypothesis that inoculation with a healthy bacterial community after antibiotic treatment can improve host immune function (*ibid*).

Several studies in mouse models have experimentally tested this hypothesis. Mice treated with antibiotics show reduced microbiome richness and higher rates of pathogen colonization and proliferation (6, 8, 10). Higher levels of labile nutrients in the gut following antibiotic treatment provide resources for the rapid and infectious growth of an invading pathogen and suggest that commensal bacteria normally limit the availability of these nutrients (6). Reestablishment or transplantation of the microbiome after antibiotic treatment can often restore colonization resistance in experimental mice (12, 13), and yet the exact mechanism behind this restoration is not yet fully understood. Restoration could result either from inherent benefits of greater community diversity in the reestablished community or from the presence of particular species in restored communities that directly compete with pathogenic bacteria.

Current research on colonization resistance focuses almost exclusively on mammalian models. Although an increasing number of studies have surveyed fish microbiomes (14–17), comparatively little is known about them, and far fewer experimental studies exist. The few studies that have addressed the role of antibiotics on fish have not focused on or fully characterized how they impact the microbiome (18, 19), and no study has documented how probiotic inoculations impact microbiome community structure. Yet fish are an important and potentially interesting model for microbiome science. Fish show wider variability in microbiome composition than mammals, differing greatly within a species across diet and environmental conditions (16, 20, 21), and the role of fish microbiomes in host health is less established. Our increasing reliance on farmed fish as an agricultural product, the persistent challenge of disease in the aquaculture industry, and the heavy reliance on prophylactic antibiotics on aquaculture farms provide significant justification for efforts to better understand how antibiotics and probiotics may impact the microbiome’s role in fish health.

We tested the hypothesis that prophylactic antibiotic administrations increase downstream mortality of the poeciliid fish *Poecilia sphenops* (black molly) after exposure to a bacterial pathogen challenge. *Poecilia* fish are a particularly interesting model for microbiome studies as they can live on a wide range of diets and under a wide range of environmental conditions, are closely related to existing models in microbiome and evolutionary studies (e.g., the Trinidadian guppy), and are highly adaptable to a laboratory environment. We also tested if the negative effects of antibiotics could be reversed without fully restoring microbiome community diversity by inoculating fish with two probiotic bacteria. We suggest that such a probiotic “rescue” from antibiotic-induced mortality would experimentally demonstrate the protective role of the fish microbiome against external pathogens, a result consistent with experiments on mammalian models.

## RESULTS

### Patterns of mortality after antibiotic and/or probiotic treatment.

**FIG 1:**
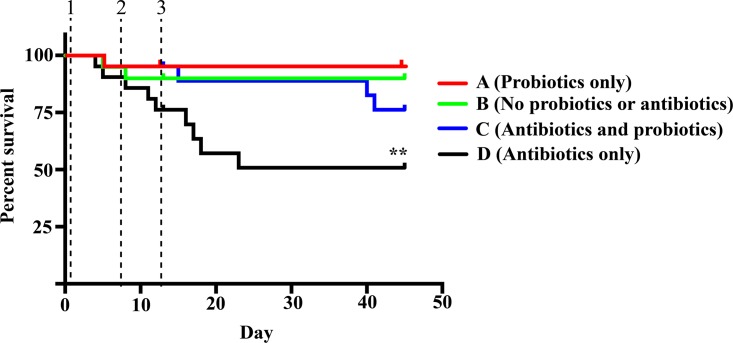
Survival curves for fish over the course of our 48-day study. The times of antibiotic administration (dashed vertical line 1), probiotics administration (dashed vertical line 2), and the bacterial challenge (dashed vertical line 3) are marked. Asterisks indicate significant results of log rank tests for comparisons to all other treatments (log rank *P* < 0.005).

Fish survivorship results at the end of our experiment (day 48) were significantly different between treatments, with treatments A (probiotics only), B (no treatment), and C (probiotic plus antibiotic) all showing greater survival than treatment D (antibiotic only; log rank [Mantel-Cox] test *P* = 0.0055) (Fig. 1). Mortalities in all treatments were spread across replicate tanks, with no significant differences between tanks within a treatment (i.e., no “tank effect”) (Table 1). Some fish mortality (between 1% and 28% depending on the treatment) was observed by day 13, prior to challenge with the pathogen *Vibrio anguillarum*, suggesting that opportunistic pathogens were primary drivers of mortality. Additional mortalities occurred after challenge only in the groups treated with antibiotics (Table 1 and Fig. 1). No significant differences in salinity or temperature were found between treatments, and nutrient measurements taken during the experimental period showed no intertreatment variation.

**TABLE 1:** Mortality data for fish in all treatments^a^

Treatment and tank	Initial no. of fish	No. of fish censored		Cumulative no. of mortalities (day 48)
		Day 13	Day 48	
A1	7	2	5	0
A2	7	1	5	1
A3	7	2	5	0
A total	21	5	15	1
				
B1	6	0	4	2
B2	6	1	5	0
B3	6	1	5	0
B total	18	2	14	2
				
C1	7	2	4	1
C2	7	1	4	2
C3	7	2	4	1
C total	21	5	12	4
				
D1	8	1	3	4
D2	8	1	4	3
D3	7	2	3	2
D total	23	4	10	9

a Treatments are indicated as follows: A, probiotic only; B, no probiotic or antibiotic; C, antibiotic and probiotic; D, antibiotic only. The data for the number of fish censored represent the number of fish removed from the experiment for sampling on the indicated day. The antibiotic treatment ran from day 1 to day 13, while probiotic treatments ran from day 9 to day 13. The pathogen challenge was given after collection on day 13. Additional fish were initially included in treatment D to account for expected mortalities.

### Presence of the pathogen *Vibrio anguillarum* in water and fish.

**FIG 2:**
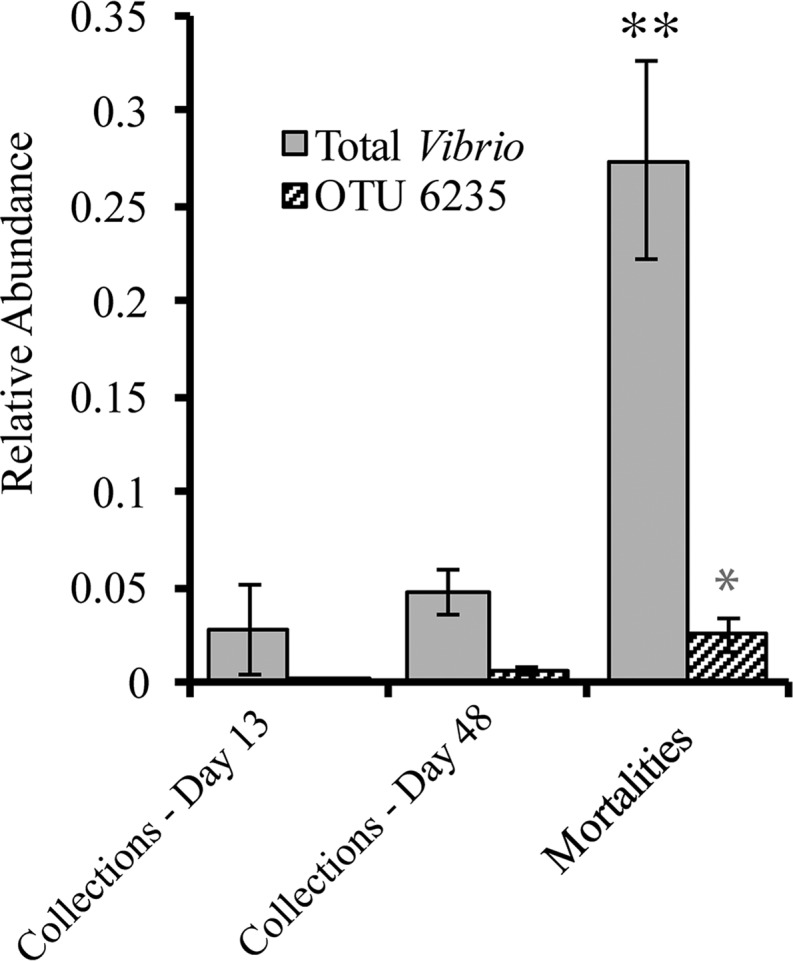
Mean relative abundances of all *Vibrio* OTUs (solid gray bars) and of our pathogen challenge *V. anguillarum* M93Sm (OTU 6235) (striped bars) within fish samples across different sampling dates and from those that died prior to collection. Double and single asterisks indicate significant differences within “Total *Vibrio*” and “OTU 6235” categories, respectively.

Operational taxonomic unit (OTU) 6235 was identified as the OTU which includes our *V. anguillarum* M93Sm-added pathogen based on 100% sequence identity. This OTU was also significantly more abundant in water samples collected 2 days after the challenge (relative abundance, 0.04 ± a standard error [SE] of 0.006) versus all other time points (0.002 ± SE 0.0005). Interestingly, although this OTU was significantly more abundant in fish that died during the experiment than in those that were collected alive, it was not the only OTU for which this was true. In fact, several other *Vibrio* OTUs were more strongly associated with fish mortality than OTU 6235 (Fig. 2). OTU 4693 in particular was highly enriched in dead fish and was 100% identical in its 16S rRNA gene V6 hypervariable region to that of the known fish pathogen *V. anguillarum* NB10 (a strain related to our pathogen) (Fig. 2). Both OTU 6235 (our pathogen) and OTU 4693 (a different *V. anguillarum*) occurred at low abundances in fish prior to the pathogen challenge, suggesting that these OTUs were already part of the fish microbiome prior to day 13 and may have been responsible for mortalities that occurred prior to our challenge. The timing of the mortalities (after antibiotic treatment but before fish were challenged) and the levels of pathogen abundance suggest that opportunistic vibrios other than our introduced pathogen may have been the cause of mortalities observed in this experiment.

### Probiotic detection in host and water microbiomes.

Several lines of evidence confirmed that OTUs 3673 and 5973 contained probiotics *B. pumilus* RI06-95 and *Phaeobacter inhibens* S4Sm, respectively. OTU 3673 had 100% identity to the reference 16S rRNA gene sequenced from RI06-95 across their region of overlap (26 bp) (NCBI GenBank accession no. KC625491.1) and was identified as corresponding to *Bacillus* using global assignment for sequence taxonomy (GAST). The representative sequence of this OTU was 100% identical to those of other *Bacillus pumilus* isolates in GenBank, as well as to those of other *Bacillus* species. Matches to multiple *Bacillus* species explain why GAST was unable to further resolve its taxonomy to the species level. Data for OTU 5973 matched at the 100% level across the V6 region of the 16S rRNA gene reference sequence from our *P. inhibens* S4Sm probiotic culture. The corresponding OTUs were identical in several species within the *Rhodobacteraceae*, including *P. gallaeciensis*, again explaining the poor resolution of the GAST-assigned taxonomy.

**FIG 3:**
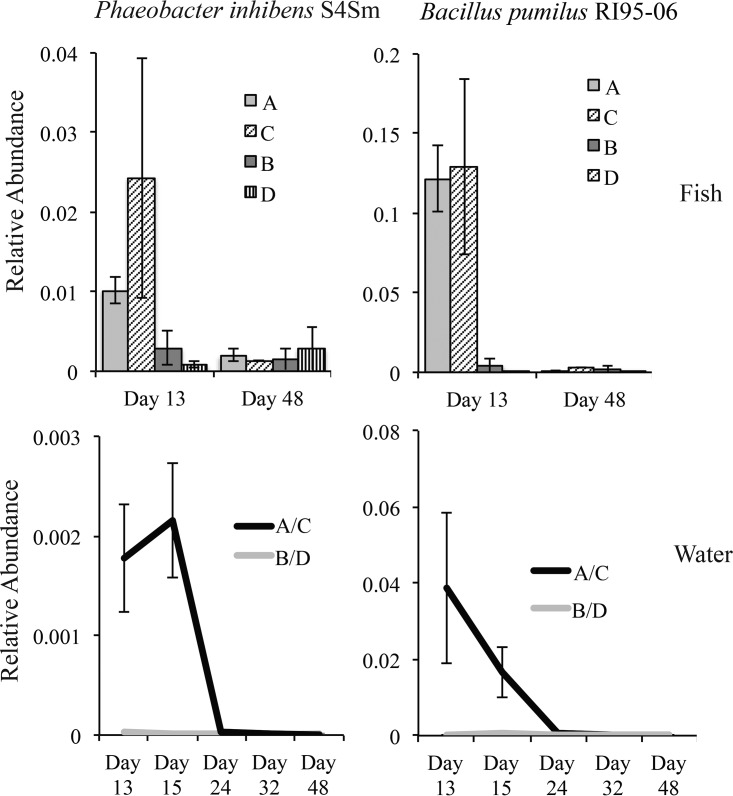
Mean relative abundances of probiotic bacteria *P. inhibens* S4Sm (OTU 5973, left) and *B. pumilus* RI06-95Sm (OTU 3673, right) in fish (top) and water (bottom) samples across sampling dates as determined by microbiome analysis. Note the *y*-axis scales differ by an order of magnitude for *B. pumilus* RI06-95Sm in water versus fish samples.

The second line of evidence that points to these OTUs as our probiotic additions is that both OTU 3673 (*P. inhibens* S4) and OTU 5973 (*B. pumilus* RI06-95) were found at significantly greater relative abundances in probiotic-treated fish samples (treatments A and C) than in the fish samples that received no probiotics (treatments B and D) on day 13 (Fig. 3). On day 13, OTU 5973 (*B. pumilus* RI06-95) occurred in fish microbiomes at a relative abundance that was an order of magnitude greater than that seen with OTU 3673 (*P. inhibens* S4), suggesting a more successful colonization of the fish. Both OTUs had decreased in their relative abundance levels by day 48, and the corresponding results were no longer significantly different from those seen with treatments B and D (Fig. 3). We also note the variance within treatment C for both OTUs on day 13, with a range of relative abundances from 0.008 to 0.08 and from 0.04 to 0.34 for OTU 3673 and OTU 5973, respectively.

Despite a trend of greater probiotic relative abundance in treatment C (antibiotic plus probiotic) than in treatment A (probiotic only), antibiotic administration did not significantly increase colonization of OTU 5973 (*B. pumilus* RI06-95) and increased colonization of OTU 3673 (*P. inhibens* S4) with only poor significance, namely, due to large variability in the success of colonization (OTU 3773 in treatment A versus treatment C, *t* test *P* = 0.048 [after Bonferroni correction]) (Fig. 3).

In water samples, both our probiotic OTU 3673 and our probiotic OTU 5973 showed a significant increase in relative abundance for day 13 samples in probiotic versus nonprobiotic treatments. Both OTUs were also found at elevated abundances in the probiotic treatments at day 15 and yet not at day 24 (on both days, only water was sampled), suggesting that these OTUs persisted in water for more than 2 days but less than 11 (Fig. 3). Fish samples showed a level of colonization by OTU 5973 (*B. pumilus* RI06-95) that was an order of magnitude greater than that shown by OTU 3673 (*P. inhibens* S4Sm), but the opposite was true for water samples, with OTU 3673 reaching a maximum relative abundance of 0.04 and OTU 5973 reaching a maximum relative abundance of 0.002 (Fig. 3).

**FIG 4:**
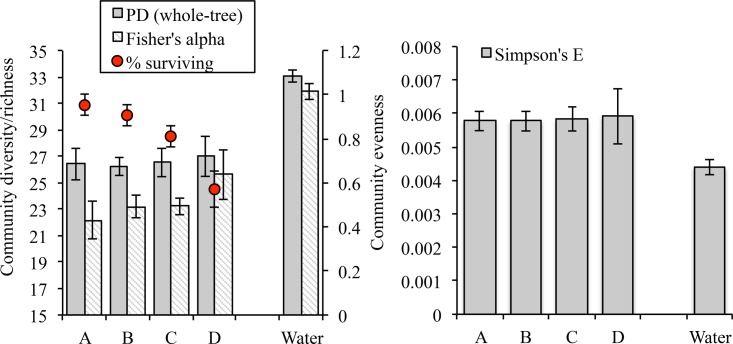
Community diversity (phylogenetic diversity [PD] and richness) and community evenness (Simpson’s E) of bacterial communities in fish and water samples collected at day 13 (directly after antibiotic treatments). No significant differences were found between treatments, although water and fish samples varied significantly with respect to both richness and evenness. The percentage of the original group of fish that survived at day 48 (final day) across tanks in each treatment is also shown in red and is overlaid across the richness data on a secondary axis.

The presence of the probiotic OTUs did not significantly influence the diversity of the communities that they colonized. Our results showed no significant differences in phylogenetic diversity of fish microbiomes across treatments directly after probiotic and antibiotic administrations (PD whole-tree test, analysis of variance [ANOVA] *P* > 0.1) (Fig. 4). Likewise, metrics of richness (observed species after rarefication and Fisher’s alpha), evenness (Simpson’s E), and species diversity (Shannon index) all showed equally nonsignificant changes.

### Microbiome community analyses.

Multivariate analyses revealed significant differences between the microbial communities in water samples and fish samples (analysis of similarity [ANOSIM] *P* < 0.0001) and between sampling dates for communities within fish (ANOSIM *P* < 0.01; see Fig. S1D in the supplemental material). Significant groupings by date occurred within water microbial communities only when the first and last sampling days (day 13 and day 48) were compared to each other (excluding intermediate sampling days). Fish that died during the experiment (referred to here as “dead”) showed a significant grouping to the exclusion of those collected alive on day 13 and day 48 (referred to here as “alive”; Fig. S1D) (ANOSIM *P* < 0.01), although this pattern was not evident in the corresponding water samples. Comparisons between the microbial communities of fish collected alive versus dead within a treatment were significant only within treatment D and treatment C (antibiotic treatments), although this was likely due to the small number of mortalities seen with treatments A and B.

10.1128/mSystems.00133-17.2 FIG S1  NMDS plots illustrating the influence of antibiotics (A), probiotics (B), treatment (C), and collection date/mortality (D) on bacterial community structure across all collection days and samples. Note that only two dates are labeled for the water collection date (D). “Yes” and “No” refer to the presence and absence of antibiotics (A) or probiotics (B). Download FIG S1, PDF file, 0.1 MB. Copyright © 2017 Schmidt et al. 2017 Schmidt et al. This content is distributed under the terms of the Creative Commons Attribution 4.0 International license.

Interestingly, administration of antibiotics (treatments C and D) and probiotics (treatments A and C) had no detectable influence on water or fish microbiomes using taxonomic-based community level analyses (ANOSIM *P* > 0.05; Fig. S1A and B) or when samples were divided into individual treatments (Fig. S1C). This pattern held true across all possible pairwise comparisons of treatments and tanks, including comparisons of antibiotic or probiotic samples within individual collection dates or treatments and within each sample type (i.e., water or fish).

At the individual OTU level, comparisons between the microbiome community compositions of fish treated with antibiotics (treatments C and D) and those not treated with antibiotics (treatments A and B) showed a surprising lack of variation. Of the 355 OTUs found in fish microbiomes after antibiotic and probiotic treatments (day 13), none showed significantly different distributions in antibiotic-treated versus non-antibiotic-treated fish at an uncorrected alpha of 0.05. Of these 355 OTUs, only 12 were significantly different in fish microbiome communities exposed to probiotics (treatments A and C) versus those without probiotics (treatments B and D), and these included the probiotic OTUs themselves.

Fish survivorship within a treatment did not correlate with any metric of microbiome diversity in that treatment on day 13 (the day that antibiotic administrations were stopped and the pathogen was added) (Fig. 4). This was true for measures of richness (Fisher’s alpha and observed species), community diversity (phylogenetic diversity and Shannon index), and community evenness (Simpson’s E). The richness of the microbial community in tank water on day 13 was also not correlated with mortality events (Fig. S2).

10.1128/mSystems.00133-17.3 FIG S2  Richness of each water sample taken on a given day, colored by treatment and ordered by tank number (i.e., first column = tank 1, last column = tank 3). Black circles indicate that a mortality event occurred near that sampling day. Download FIG S2, PDF file, 0.1 MB. Copyright © 2017 Schmidt et al. 2017 Schmidt et al. This content is distributed under the terms of the Creative Commons Attribution 4.0 International license.

### Taxonomic composition of black molly microbiome communities.

**FIG 5:**
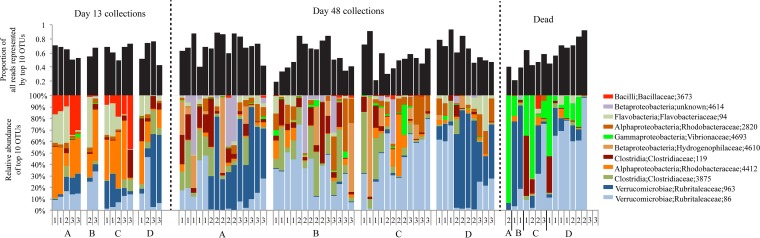
Relative abundances of the top 10 most abundant OTUs across all fish samples are shown along with the data from the probiotic *B. pumilus* RI06-95 (OTU 3673) (bottom). Also shown is the proportion of all reads that the top 10 OTUs represent (top). Treatment and tank number within treatments are shown below. Note that the samples that were collected dead varied in their collection date and that data are missing from two fish from tank D3 (far right).

The top 10 most abundant OTUs found in black molly microbiomes from this study, as measured by mean levels of relative abundance, included six classes across three phyla: *Clostridia* and *Bacilli* (*Firmicutes*); *Flavobacterium* (*Bacteroidetes*); *Betaproteobacteria*,* Alphaproteobacteria*, and *Gammaproteobacteria* (*Proteobacteria*); and *Verrucomicrobia* (*Verrucomicrobia*) (Fig. 5 and Text S1 in the supplemental material). Two OTUs of *Verrucomicrobia* of the *Rubritalea* genus were the most abundant OTUs in our data set and represented a combined mean relative abundance of 0.29 (SE ± 0.013), which reached as high as 0.90 in a single sample (OTU 86 and OTU 963; Fig. 5). The two *Verrucomicrobia* OTUs also appeared to be negatively correlated with one another, with only one of the two dominant in a given fish (Fig. S3). Bacterial communities in tank water were dominated by OTUs from three families, *Rhodobacteraceae*, *Flavobacteriaceae*, and *Alteromonadaceae*, representing a cumulative average relative abundance of nearly 50% (Fig. S4 and Text S1).

10.1128/mSystems.00133-17.1 TEXT S1  Supplemental results and methods. Download TEXT S1, DOCX file, 0.1 MB. Copyright © 2017 Schmidt et al. 2017 Schmidt et al. This content is distributed under the terms of the Creative Commons Attribution 4.0 International license.

10.1128/mSystems.00133-17.4 FIG S3  Relative abundances of the two most abundant OTUs across all fish samples. Each point represents a single sample, with its relative abundance plotted from each OTU. The two OTUs show a strongly negative correlation to one another, a pattern driven by tank membership. Download FIG S3, PDF file, 0.05 MB. Copyright © 2017 Schmidt et al. 2017 Schmidt et al. This content is distributed under the terms of the Creative Commons Attribution 4.0 International license.

10.1128/mSystems.00133-17.5 FIG S4  Relative abundances of the top 10 most abundant OTUs in each water sample across treatments and time. Each tank had 5 samples corresponding to the five sampling points (days 12, 15, 24, 32, and 48). More than 10 OTUs are shown since not all treatments had the same 10 top OTUs. OTUs are colored by family, with our *Phaeobacter* sp. strain S4 (OTU 5973) shown in bright red. Download FIG S4, PDF file, 0.3 MB. Copyright © 2017 Schmidt et al. 2017 Schmidt et al. This content is distributed under the terms of the Creative Commons Attribution 4.0 International license.

## DISCUSSION

Prophylactic antibiotics in the aquaculture and ornamental fish industries are intended to prevent disease and are administered widely where vaccination is not feasible (e.g., in the case of invertebrate species, in hatcheries, or when no vaccines are available) or cost prohibitive (22, 23). Our study found that prophylactic antibiotic administration prior to pathogen exposure increased rates of mortality in fish across replicate treatment tanks, suggesting a negative long-term effect of the antibiotic administrations on disease resistance. Further, our study found that colonization by two candidate probiotic species after antibiotic treatment can prevent antibiotic-induced mortalities without influencing the overall community structure or diversity of the microbiome. No study had demonstrated this effect before in an aquatic organism.

Although our data corroborate previous research in mammalian models indicating that antibiotic administration increases downstream mortality (6, 8, 10), limitations of our data set prevent any determination of causal mechanism. Several microbe-independent mechanisms may have increased mortality in our antibiotic-only treatment (treatment D), including a directly toxic effect of the antibiotics or an antibiotic-induced increase in pathogen virulence. We therefore emphasize that the mechanism of increased mortality after antibiotic treatment in our study remains unknown. However, our study clearly demonstrated that inoculation with two strains of probiotic bacteria reverses the negative impact of antibiotic administrations. Because both probiotics were live bacteria and because all experimental procedures were identical between treatments except the addition of these species, the reversal of antibiotic-induced mortality was very likely a result of either direct or indirect positive microbial processes. We further demonstrate that successful colonization of the probiotics in the fish microbiome community did not significantly change the overall structure or diversity of that community, despite changes to the host phenotype after antibiotic administrations (mortality rates). This implies that subtle changes to microbiome composition can influence its function without wholesale changes to community diversity, a result that contrasts with existing hypotheses related to the nature of microbiome diversity, colonization resistance, and health (24).

Interestingly, in the plant communities where the hypothesis that increased diversity correlates with increased colonization resistance (often termed “biotic resistance”) was first presented, the susceptibility to invasive species rarely correlates with native species richness, and other factors such as ecosystem composition, productivity, habitat and environmental heterogeneity, and colonization rates can all influence the success of invasive species (4, 5, 25, 26). Taken together, these conclusions present the possibility that colonization resistance can be manipulated independently of overall community diversity using only small additions to the composition of a host microbiome. We strongly emphasize, however, that it is still possible that reductions in diversity can also lead to reductions in colonization resistance and vice versa, and in this case, colonization resistance could be manipulated both by shifts in overall community diversity and by subtle changes in microbiome composition.

### Antibiotic administrations may have facilitated the activity of opportunistic *Vibrio* pathogens.

Pathogens found in the ambient environment of our experimental system may have played a more central role in our mortality results than the *V. anguillarum* M93Sm challenge itself. Several of our results support this conclusion. First, the influence of antibiotic treatments on mortality rates was seen days before the pathogen challenge. Second, although all treatments received a challenge, only the treatment D group had significant mortality (suggesting that the challenge with *V. anguillarum* M93Sm strain was ineffective). Lastly, the abundances of environmental vibrios other than *V. anguillarum* M93Sm were significantly enriched in the fish that died during the experiment, suggesting opportunistic infections in antibiotic-treated fish. This trend was observed across all three replicate tanks in treatment D and therefore did not represent a single outbreak or “tank effect.” Observations of *Vibrio* ecology also support this conclusion. Vibrios are known opportunistic pathogens in fish (27) and are thought to be “r-strategists” capable of rapid growth and virulence in disturbed microbial communities (28, 29). Furthermore, the disruption of native microbial communities with prophylactic antibiotic use in advance of *Vibrio* outbreaks is thought to worsen mortality in the aquaculture industry (29, 30), a suggestion supported by our experimental results.

Interestingly, our probiotic treatments mitigated increased mortality after antibiotic administrations. The mechanism behind this impact remains unclear, but we note that both of the probiotic species used in this study have been shown to inhibit *Vibrio* growth *in vitro* (31–33). *P. inhibens* is a particularly strong inhibitor of *Vibrio* growth and its use as an effective aquaculture probiotic has been advocated (34, 35). The strain produces the quorum quenching molecule n-acyl homoserine lactone (AHL) and the antibiotic tropodithietic acid (TDA), both of which are highly effective against *Vibrio* virulence and growth (31, 36, 37). One scenario that may explain our results works as follows: antibiotic administrations disturb the fish microbiome (reducing colonization resistance), favoring the growth of opportunistic *Vibrio* pathogens, which in turn increases fish mortality in antibiotic-only tanks (treatment D). In antibiotic-plus-probiotic tanks (treatment C), competition with or inhibition of *Vibrio* by our two probiotic species removes this advantage. Additional experimental research should be conducted in simplified systems, including gnotobiotic zebrafish (38), to assess the competitive interaction between these two probiotic species and *Vibrio* pathogens.

### Sample collection techniques likely obscured a microbiome-antibiotic signal.

Surprisingly, our 16S rRNA amplicon sequencing data did not reveal an influence of antibiotics on fish microbiome communities. This result was evident across a wide range of analysis metrics, including several multivariate community similarity metrics and distributions of individual OTUs. Of the 355 OTUs found in fish microbiomes after antibiotic treatments (day 13), none showed significantly different levels of relative abundance in treated or untreated fish at an uncorrected alpha of 0.05. At this alpha level, nearly 18 OTUs were expected to show significantly different distributions by chance alone. Equally surprising was the lack of influence on community alpha diversity from antibiotic administrations as neither species richness nor evenness, measured using both phylogenetic metrics and species counts, varied between any of the treatments in this study.

Both our alpha and beta diversity results stand in contrast to previous studies on the role of antibiotics in terrestrial animals. A clear signal of antibiotic drug administration is evident in the microbiomes of mice (7), swine (39), rats (40), and humans (41). Several studies have even found an impact on the abundance and composition of fish microbiome communities after antibiotic treatment (18, 19), although we note that no such study has used culture-independent high-throughput sequencing.

In light of this previous literature and of the significant impact of antibiotics on mortality in our data, the lack of antibiotic impact on our microbiomes is surprising. We suggest that our sampling procedure, in which whole fish carcasses were processed for microbial analysis, may have contributed to this result. It is possible that microbial habitats in the fish that were not exposed to adequate concentrations of the antibiotic but that contained extremely high concentrations of bacteria (e.g., in the lower intestine [42]) overwhelmed any signal from habitats which both were impacted by the antibiotic and played a role in colonization resistance (e.g., gills or skin). We therefore must emphasize that our results do not demonstrate resilience of the fish microbiome community with respect to antibiotic treatment, a conclusion that would stand in stark contrast to the clear impact of the antibiotic treatment on mortality in our data and to a wealth of existing literature.

## MATERIALS AND METHODS

### Bacterial strains.

We obtained *V. anguillarum* M93Sm (a spontaneous mutant of M93 resistant to streptomycin), a bacterial pathogen of finfish and shellfish, from D. Nelson at the University of Rhode Island and maintained it according to the method previously described by Zhao et al. (31). We prepared daily cultures of the probiotic bacterial strains *P. inhibens* S4Sm and *B. pumilus* RI06-95Sm in Luria-Bertani broth containing 1.5 mg/liter streptomycin and 2% NaCl. We determined cell concentrations via serial dilution and spotting on agar plates following established protocols (31, 32, 43). All three bacterial strains were well known to us prior to their use in this study, and careful documentation of their growth rates, genome sequences, and virulence is outlined elsewhere (31, 32).

### Experimental design and sample collection.

We purchased *P. sphenops* black mollies of between 20 and 30 mm in length from a wholesale supplier (PetSolutions, Beavercreek, OH) that maintained fish at between 0.0 ppt and 2.0 ppt in mixed-species aquaria. This species is a particularly interesting choice for microbiome experiments as it can live across a wide range of environmental conditions and diets, allowing these variables to be manipulated while holding host taxonomy constant (see reference 16). It also gives live birth, is closely related to a range of existing model poeciliid fish used in microbiome and evolutionary studies (e.g., Trinidadian guppy [44]), and is highly adaptable to a laboratory environment.

**FIG 6:**
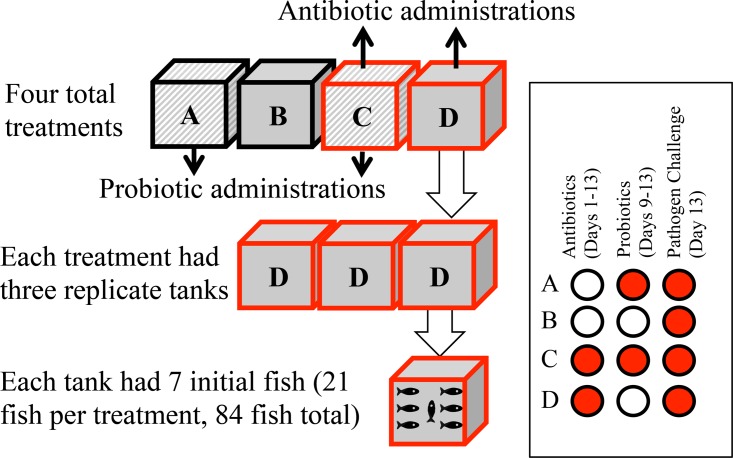
Study design, showing overall treatment layout (top), tank replication within each treatment (middle), and fish replication within each tank (bottom). Shown at right is a layout of antibiotic and probiotic administrations for each treatment.

After purchase, we acclimated ~150 fish to 30 ppt by adding sterilized seawater (UV treated and 0.22-μm-pore-size filtered) at no more than 1 ppt per day over a 45-day period. All fish were acclimated together. We then randomly selected all fish of similar sizes and placed them into four treatment environments, each consisting of three replicate 125-liter tanks with six, seven, or eight fish in each tank (Fig. 6). Eight fish were subjected to treatment D, versus only 6 to treatment B, to account for higher predicted mortalities with treatment D versus treatment B (control). Five days prior to the addition of fish, we placed tanks in a shared water bath of flowing seawater at 20°C to control temperature; filled each tank with filtered and UV-treated seawater from Narragansett Bay, RI; and fitted them with a recirculating aquarium biofilter and a charcoal filter with aeration through an airstone. Water quality (temperature, salinity, and total ammonia and nitrogen) was determined twice per week throughout the experiment using a Pro30 sensor (YSI, Inc., Yellow Springs, OH) and an API saltwater test kit (API, Blacksburg, VT).

Treatments included (A) probiotics only; (B) no treatment (control); (C) antibiotics plus probiotics; and (D) antibiotics only. Antibiotics were administered daily to the antibiotic-treated groups from day 1 to day 13. We administered probiotics daily for 5 days from day 8 to day 13, such that the tail end of the antibiotic administrations overlapped with the probiotic administrations (Fig. 6). Note that both probiotic species were resistant to our antibiotic treatment (see below). After antibiotic and probiotic administrations (day 13), we collected and euthanized subsets of fish from each tank using 10 mg/ml MS-222 according to IACUC protocols. After euthanization, fish were transferred into tubes with 40 ml of sterile 1× phosphate-buffered saline (PBS), subjected to light vortex mixing to remove unassociated microbes, removed from the PBS, and stored in sterile bags at −20°C for microbiome analysis.

All treatments then received a challenge of *V. anguillarum* M93Sm followed by daily mortality assessments (see below for challenge protocols). We collected each dead fish as described above immediately after it was discovered (and never more than 12 h later) and euthanized all remaining fish after 48 days as outlined above. We extracted genomic DNA (gDNA) according to the method described by Schmidt et al. (16) by rinsing and homogenizing the undissected fish carcass. Homogenizing the entire fish was done to potentially capture a systemic effect of the probiotic exposure and pathogen challenge (45). We monitored water quality routinely and changed water as needed.

Over the 48-day experiment, we sampled tank water five times to characterize bacterial communities at the following time points: immediately prior to the pathogen challenge (day 13); 2 days after the challenge (day 15); and then subsequently on days 24, 32, and 49. We stored all water and fish tissue samples at −20°C until genomic DNA extraction (performed within 3 months). Microbial communities were collected from the tank water by filtering 1 liter of water through a 0.2 μm-pore-size Sterivex filter (Millipore, Billerica, MA) (16) (see Text S1 in the supplemental material).

### Antibiotic, probiotic, and pathogen administrations.

We administered antibiotics (streptomycin sulfate; final concentration of 200 μg/ml) by removing fish from tanks and placing them in buckets for 90 to 120 min with 1.5 liters of sterile seawater aerated with in-line air pumps. Streptomycin was chosen as the antibiotic because our probiotic and pathogen cultures had been selected for streptomycin resistance (31, 32). Fresh stocks of streptomycin sulfate solution were prepared every other day and diluted directly into bucket water before each administration. We fed fish TetraMin (API, Blacksburg, VA) tropical fish feed during antibiotic administrations in an attempt to facilitate antibiotic ingestion. After antibiotic administration, we rinsed fish in filtered-sterile seawater and placed them back into their respective tanks. Probiotics and pathogens were administered in a similar fashion, adding 1 × 10^5^ CFU/ml final concentrations of *P. inhibens* S4Sm and *B. pumilus* RI06-95Sm to the water of each bucket. Probiotic doses were chosen based on previous experiments performed in our laboratory that showed maximum protection of larval shellfish against *Vibrio* pathogens at these relatively low concentrations. Note that the fish were handled in the same way each day in all treatments, such that control fish were also transferred to buckets but not given drug or probiotic treatments. Fish were challenged in 1.5-liter water in sterile buckets by exposing the fish to 1 × 10^7^ CFU/ml of *V. anguillarum* M93Sm for 120 min with food and aeration. The entire contents of the bucket were then poured directly into treatment tanks.

### Library preparation, sequencing, and bioinformatics analyses.

We sequenced the V6 hypervariable region of the bacterial 16S rRNA gene using a custom 2-step “fusion primer” PCR amplification. First, we performed an initial 20-cycle PCR in triplicate using a cocktail of standard forward and reverse universal bacterial primers (967F and 1064R); we then amplified this product in a second 10-cycle PCR using primers with Illumina HiSeq adaptors and barcodes attached to their 5′ end. Our fusion PCR protocols and primer sequences are further explained elsewhere (https://vamps.mbl.edu/resources/primers.php). Paired-end sequencing was conducted at the WM Keck Ecological and Evolutionary Genetics Facility at the Marine Biological Laboratory (MBL) using an Illumina HiSeq 1000 system and generated 100-bp reads with 100% overlap of reads 1 (forward) and 2 (reverse). Quality filtering and error removal followed standard protocols at the MBL’s Bay Paul Center that remove reads where forward and reverse sequences do not match perfectly (46).

OTU clustering was done using minimum entropy decomposition (MED) to cluster sequences into MED nodes (47). After clustering, we used the most common sequence in a given OTU as a representative sequence for that OTU and used the GAST pipeline (48) to assign taxonomy to each representative sequence. Finally, we uploaded our resulting MED matrix to the VAMPS (visualization and analysis of microbial population structure) interface and normalized the entire matrix to the total (relative abundance). Further public analysis and exploration of these data are possible on the VAMPS website (https://vamps.mbl.edu/) using software under the project name VTS_PROnodes. All original sequence files and minimum information about marker gene sequence (MIMARKS) compliant data (49) were deposited in NCBI’s Sequence Read Archive (see below). MIMARKS metadata tables are also attached here as a supplementary file (see Table S1 in the supplemental material).

10.1128/mSystems.00133-17.6 TABLE S1  MIMARKS table with metadata for all data sets. Download TABLE S1, XLSX file, 0.1 MB. Copyright © 2017 Schmidt et al. 2017 Schmidt et al. This content is distributed under the terms of the Creative Commons Attribution 4.0 International license.

### Statistical analyses.

We performed fish survival analysis using the Mantel-Cox log rank test as implemented in Prism version 6.0 (GraphPad Software, Inc., La Jolla, CA, USA). Fish collected on day 13 and 48 were considered censored (i.e., removed from the experiment for sampling on the indicated day), while all others were scored as mortalities. In order to determine the potential role of pseudoreplication due to tank effects, we assessed if any significant mortality differences could be found between tanks within a treatment using pairwise Mantel-Cox log rank tests.

Analyses of community richness employed Qiime v. 1.5 (50) with normalized MED OTU tables (see above). We estimated richness using Chao1 and Fisher’s alpha and estimated evenness using Simpson’s E (51) as implemented in Qiime’s *alpha_diversity.py* script. We determined phylogenetic diversity (PD) by first aligning all OTU representative sequences using MUSCLE (52) and then constructing a Randomized Axelerated Maximum Likelihood (RAxML) phylogeny (53). Pairwise *t* tests performed with Bonferroni correction for multiple comparisons determined significant differences between treatments for probiotic and pathogen relative abundance.

For community-level comparisons, we used single-factor permutation-based multivariate analysis of similarity (ANOSIM) implemented in PrimerE v6.1 and 9,999 permutations on a MED OTU-derived Bray-Curtis similarity matrix, with Bonferroni corrections for multiple comparisons. We visualized results using nonmetric multidimentional scaling plots (NMDS) and covariance ellipsoids using the betadisper{vegan} function in R (54), as implemented in the oligotyping pipeline (55).

### Accession number(s).

All original sequence files and minimum information about marker gene sequence (MIMARKS) compliant data (49) were deposited in NCBI’s Sequence Read Archive under BioProject accession no. PRJNA362181.

## References

[1] Turnbaugh PJ, Ley RE, Hamady M, Fraser-Liggett CM, Knight R, Gordon JI 2007 The human microbiome project. Nature 449:804–810. doi:10.1038/nature06244. 17943116 PMC3709439

[2] Cain K, Swan C 2010 Barrier function and immunology, p 118–125. *In* Grosell M, Farrell AP, Brauner CJ (ed), Fish physiology: the multifunctional gut of fish, vol 30 Academic Press, San Diego, CA. doi:10.1016/S1546-5098(10)03003-7.

[3] Elton C 1958 The ecology on invasions by animals and plants. Chapman & Hall, London, United Kingdom.

[4] Levine JM, Adler PB, Yelenik SG 2004 A meta-analysis of biotic resistance to exotic plant invasions. Ecol Lett 7:975–989. doi:10.1111/j.1461-0248.2004.00657.x.

[5] Fridley JD, Stachowicz JJ, Naeem S, Sax DF, Seabloom EW, Smith MD, Stohlgren TJ, Tilman D, Von Holle B 2007 The invasion paradox: reconciling pattern and process in species invasions. Ecology 88:3–17. doi:10.1890/0012-9658(2007)88[3:TIPRPA]2.0.CO;2. 17489447

[6] Ng KM, Ferreyra JA, Higginbottom SK, Lynch JB, Kashyap PC, Gopinath S, Naidu N, Choudhury B, Weimer BC, Monack DM, Sonnenburg JL 2013 Microbiota-liberated host sugars facilitate post-antibiotic expansion of enteric pathogens. Nature 502:96–99. doi:10.1038/nature12503. 23995682 PMC3825626

[7] Sekirov I, Tam NM, Jogova M, Robertson ML, Li Y, Lupp C, Finlay BB 2008 Antibiotic-induced perturbations of the intestinal microbiota alter host susceptibility to enteric infection. Infect Immun 76:4726–4736. doi:10.1128/IAI.00319-08. 18678663 PMC2546810

[8] Theriot CM, Koenigsknecht MJ, Carlson PE, Hatton GE, Nelson AM, Li B, Huffnagle GB, Li JZ, Young VB 2014 Antibiotic-induced shifts in the mouse gut microbiome and metabolome increase susceptibility to *Clostridium difficile* infection. Nat Commun 5:3114. doi:10.1038/ncomms4114. 24445449 PMC3950275

[9] Youngster I, Sauk J, Pindar C, Wilson RG, Kaplan JL, Smith MB, Alm EJ, Gevers D, Russell GH, Hohmann EL 2014 Fecal microbiota transplant for relapsing *Clostridium difficile* infection using a frozen inoculum from unrelated donors: a randomized, open-label, controlled pilot study. Clin Infect Dis 58:1515–1522. doi:10.1093/cid/ciu135. 24762631 PMC4017893

[10] Chang JY, Antonopoulos DA, Kalra A, Tonelli A, Khalife WT, Schmidt TM, Young VB 2008 Decreased diversity of the fecal microbiome in recurrent *Clostridium difficile*-associated diarrhea. J Infect Dis 197:435–438. doi:10.1086/525047. 18199029

[11] Aas J, Gessert CE, Bakken JS 2003 Recurrent *Clostridium difficile* colitis: case series involving 18 patients treated with donor stool administered via a nasogastric tube. Clin Infect Dis 36:580–585. doi:10.1086/367657. 12594638

[12] Pamer EG 2016 Resurrecting the intestinal microbiota to combat antibiotic-resistant pathogens. Science 352:535–538. doi:10.1126/science.aad9382. 27126035 PMC4984266

[13] Buffie CG, Pamer EG 2013 Microbiota-mediated colonization resistance against intestinal pathogens. Nat Rev Immunol 13:790–801. doi:10.1038/nri3535. 24096337 PMC4194195

[14] Givens CE, Ransom B, Bano N, Hollibaugh JT 2015 Comparison of the gut microbiomes of 12 bony fish and 3 shark species. Mar Ecol Prog Ser 518:209–223. doi:10.3354/meps11034.

[15] Llewellyn MS, Boutin S, Hoseinifar SH, Derome N 2014 Teleost microbiomes: the state of the art in their characterization, manipulation and importance in aquaculture and fisheries. Front Microbiol 5:207. doi:10.3389/fmicb.2014.00207. 24917852 PMC4040438

[16] Schmidt VT, Smith KF, Melvin DW, Amaral-Zettler LA 2015 Community assembly of a euryhaline fish microbiome during salinity acclimation. Mol Ecol 24:2537–2550. doi:10.1111/mec.13177. 25819646

[17] Smith CC, Snowberg LK, Gregory Caporaso J, Knight R, Bolnick DI 2015 Dietary input of microbes and host genetic variation shape among-population differences in stickleback gut microbiota. ISME J 9:2515–2526. doi:10.1038/ismej.2015.64. 25909977 PMC4611514

[18] Austin B, Al-Zahrani AMJ 1988 The effect of antimicrobial compounds on the gastrointestinal microflora of rainbow trout, *Salmo gairdneri*. J Fish Biol 33:1–14. doi:10.1111/j.1095-8649.1988.tb05444.x.

[19] Navarrete P, Mardones P, Opazo R, Espejo R, Romero J 2008 Oxytetracycline treatment reduces bacterial diversity of intestinal microbiota of Atlantic salmon. J Aquat Anim Health 20:177–183. doi:10.1577/H07-043.1. 18942594

[20] Bolnick DI, Snowberg LK, Hirsch PE, Lauber CL, Knight R, Caporaso JG, Svanbäck R 2014 Individuals’ diet diversity influences gut microbial diversity in two freshwater fish (threespine stickleback and Eurasian perch). Ecol Lett 17:979–987. doi:10.1111/ele.12301. 24847735 PMC4084827

[21] Schmidt V, Amaral-Zettler L, Davidson J, Summerfelt S, Good C 2016 Influence of fishmeal-free diets on microbial communities in Atlantic salmon (*Salmo salar*) recirculation aquaculture systems. Appl Environ Microbiol 82:4470–4481. doi:10.1128/AEM.00902-16. 27129964 PMC4984271

[22] Yanong R 2010 Use of antibiotics in ornamental fish aquaculture. Publication #CIR 84 University of Florida Institute of Food and Agricultural Sciences (UF/IFAS), Gainesville, FL http://edis.ifas.ufl.edu/fa084.

[23] Cabello FC 2006 Heavy use of prophylactic antibiotics in aquaculture: a growing problem for human and animal health and for the environment. Environ Microbiol 8:1137–1144. doi:10.1111/j.1462-2920.2006.01054.x. 16817922

[24] Human Microbiome Project Consortium 2012 Structure, function and diversity of the healthy human microbiome. Nature 486:207–214. doi:10.1038/nature11234. 22699609 PMC3564958

[25] Shurin JB 2000 Dispersal limitation, invasion resistance, and the structure of pond zooplankton communities. Ecology 81:3074–3086. doi:10.1890/0012-9658(2000)081[3074:DLIRAT]2.0.CO;2.

[26] Jiang L, Morin PJ 2004 Productivity gradients cause positive diversity-invasibility relationships in microbial communities. Ecol Lett 7:1047–1057. doi:10.1111/j.1461-0248.2004.00660.x.

[27] Austin B, Austin D 2007 Bacterial fish pathogens; diseases of farmed and wild fish, 4th ed. Praxis Publishing, Chichester, United Kingdom.

[28] Schmidt VT, Reveillaud J, Zettler E, Mincer TJ, Murphy L, Amaral-Zettler LA 2014 Oligotyping reveals community level habitat selection within the genus *Vibrio*. Front Microbiol 5:563. doi:10.3389/fmicb.2014.00563. 25431569 PMC4230168

[29] De Schryver P, Vadstein O 2014 Ecological theory as a foundation to control pathogenic invasion in aquaculture. ISME J 8:2360–2368. doi:10.1038/ismej.2014.84. 24892581 PMC4260705

[30] Defoirdt T 2016 Implications of ecological niche differentiation in marine bacteria for microbial management in aquaculture to prevent bacterial disease. PLOS Pathog 12:e1005843. doi:10.1371/journal.ppat.1005843. 27832154 PMC5104322

[31] Zhao W, Dao C, Karim M, Gomez-Chiarri M, Rowley D, Nelson DR 2016 Contributions of tropodithietic acid and biofilm formation to the probiotic activity of *Phaeobacter inhibens*. BMC Microbiol 16:1. doi:10.1186/s12866-015-0617-z. 26728027 PMC4700733

[32] Karim M, Zhao W, Rowley D, Nelson D, Gomez-Chiarri M 2013 Probiotic strains for shellfish aquaculture: protection of eastern oyster, *Crassostrea virginica*, larvae and juveniles against bacterial challenge. J Shellfish Res 32:401–408. doi:10.2983/035.032.0220.

[33] Vaseeharan B, Ramasamy P 2003 Control of pathogenic *Vibrio* spp. by *Bacillus subtilis* BT23, a possible probiotic treatment for black tiger shrimp *Penaeus monodon*. Lett Appl Microbiol 36:83–87. doi:10.1046/j.1472-765X.2003.01255.x. 12535126

[34] Grotkjær T, Bentzon-Tilia M, D’Alvise P, Dierckens K, Bossier P, Gram L 2016 *Phaeobacter inhibens* as probiotic bacteria in non-axenic artemia and algae cultures. Aquaculture 462:64–69. doi:10.1016/j.aquaculture.2016.05.001.

[35] D’Alvise PW, Lillebø S, Wergeland HI, Gram L, Bergh Ø 2013 Protection of cod larvae from vibriosis by *Phaeobacter* spp.: a comparison of strains and introduction times. Aquaculture 384–387:82–86. doi:10.1016/j.aquaculture.2012.12.013.

[36] Bertrand G, Olivier L, Jean-louis N, Philippe M, Marie-lou B, Réjean T 2014 Effect of the probiotic strain *Phaeobacter gallaeciensis* after bacterial challenge on the complete larval development of *Pecten maximus*. Aquat Living Resour 27:27–34. doi:10.1051/alr/2014005.

[37] Pande GSJ, Scheie AA, Benneche T, Wille M, Sorgeloos P, Bossier P, Defoirdt T 2013 Quorum sensing-disrupting compounds protect larvae of the giant freshwater prawn *Macrobrachium rosenbergii* from *Vibrio harveyi* infection. Aquaculture 406–407:121–124. doi:10.1016/j.aquaculture.2013.05.015.

[38] Pham LN, Kanther M, Semova I, Rawls JF 2008 Methods for generating and colonizing gnotobiotic zebrafish. Nat Protoc 3:1862–1875. doi:10.1038/nprot.2008.186. 19008873 PMC2596932

[39] Looft T, Johnson TA, Allen HK, Bayles DO, Alt DP, Stedtfeld RD, Sul WJ, Stedtfeld TM, Chai B, Cole JR, Hashsham SA, Tiedje JM, Stanton TB 2012 In-feed antibiotic effects on the swine intestinal microbiome. Proc Natl Acad Sci U S A 109:1691–1696. doi:10.1073/pnas.1120238109. 22307632 PMC3277147

[40] Manichanh C, Reeder J, Gibert P, Varela E, Llopis M, Antolin M, Guigo R, Knight R, Guarner F 2010 Reshaping the gut microbiome with bacterial transplantation and antibiotic intake. Genome Res 20:1411–1419. doi:10.1101/gr.107987.110. 20736229 PMC2945190

[41] Dethlefsen L, Huse S, Sogin ML, Relman DA 2008 The pervasive effects of an antibiotic on the human gut microbiota, as revealed by deep 16S rRNA sequencing. PLoS Biol 6:e280. doi:10.1371/journal.pbio.0060280. 19018661 PMC2586385

[42] Grosell M, Farrell AP, Brauner CJ (ed) 2011 Fish physiology: the multifunctional gut of fish, vol. 30 Academic Press, San Diego, CA.

[43] Sohn S, Lundgren KM, Tammi K, Karim M, Smolowitz R, Nelson DR, Rowley DC, Gómez-Chiarri M 2016 Probiotic strains for disease management in hatchery larviculture of the eastern oyster *Crassostrea virginica*. J Shellfish Res 35:307–317. doi:10.2983/035.035.0205.

[44] Sullam KE, Rubin BE, Dalton CM, Kilham SS, Flecker AS, Russell JA 2015 Divergence across diet, time and populations rules out parallel evolution in the gut microbiomes of Trinidadian guppies. ISME J 9:1508–1522. doi:10.1038/ismej.2014.231. 25575311 PMC4478690

[45] O’Toole R, von Hofsten J, Rosqvist R, Olsson PE, Wolf-Watz H 2004 Visualisation of zebrafish infection by GFP-labelled *Vibrio anguillarum*. Microb Pathog 37:41–46. doi:10.1016/j.micpath.2004.03.001. 15194159

[46] Eren AM, Vineis JH, Morrison HG, Sogin ML 2013 A filtering method to generate high quality short reads using Illumina paired-end technology. PLoS One 8:e66643. doi:10.1371/journal.pone.0066643. 23799126 PMC3684618

[47] Eren AM, Morrison HG, Lescault PJ, Reveillaud J, Vineis JH, Sogin ML 2015 Minimum entropy decomposition: unsupervised oligotyping for sensitive partitioning of high-throughput marker gene sequences. ISME J 9:968–979. doi:10.1038/ismej.2014.195. 25325381 PMC4817710

[48] Huse SM, Dethlefsen L, Huber JA, Mark Welch D, Welch DM, Relman DA, Sogin ML 2008 Exploring microbial diversity and taxonomy using SSU rRNA hypervariable tag sequencing. PLoS Genet 4:e1000255. doi:10.1371/journal.pgen.1000255. 19023400 PMC2577301

[49] Yilmaz P, Kottmann R, Field D, Knight R, Cole JR, Amaral-Zettler L, Gilbert JA, Karsch-Mizrachi I, Johnston A, Cochrane G, Vaughan R, Hunter C, Park J, Morrison N, Rocca-Serra P, Sterk P, Arumugam M, Bailey M, Baumgartner L, Birren BW, Blaser MJ, Bonazzi V, Booth T, Bork P, Bushman FD, Buttigieg PL, Chain PSG, Charlson E, Costello EK, Huot-Creasy H, Dawyndt P, DeSantis T, Fierer N, Fuhrman JA, Gallery RE, Gevers D, Gibbs RA, San Gil I, Gonzalez A, Gordon JI, Guralnick R, Hankeln W, Highlander S, Hugenholtz P, Jansson J, Kau AL, Kelley ST, Kennedy J, Knights D, Koren O, et al. 2011 Minimum information about a marker gene sequence (MIMARKS) and minimum information about any (x) sequence (MIxS) specifications. Nat Biotechnol 29:415–420. doi:10.1038/nbt.1823. 21552244 PMC3367316

[50] Caporaso JG, Kuczynski J, Stombaugh J, Bittinger K, Bushman FD, Costello EK, Fierer N, Peña AG, Goodrich JK, Gordon JI, Huttley GA, Kelley ST, Knights D, Koenig JE, Ley RE, Lozupone CA, McDonald D, Muegge BD, Pirrung M, Reeder J, Sevinsky JR, Turnbaugh PJ, Walters WA, Widmann J, Yatsunenko T, Zaneveld J, Knight R 2010 QIIME allows analysis of high-throughput community sequencing data. Nat Methods 7:335–336. doi:10.1038/nmeth.f.303. 20383131 PMC3156573

[51] Magurran A 2004 Measurning biological diversity. Blackwell Publishing Science Ltd, Malden, MA.

[52] Edgar RC 2004 MUSCLE: multiple sequence alignment with high accuracy and high throughput. Nucleic Acids Res 32:1792–1797. doi:10.1093/nar/gkh340. 15034147 PMC390337

[53] Stamatakis A 2006 RAxML-VI-HPC: maximum likelihood-based phylogenetic analyses with thousands of taxa and mixed models. Bioinformatics 22:2688–2690. doi:10.1093/bioinformatics/btl446. 16928733

[54] Oksanen J, Blanchet FG, Kindt R, Legendre P, O’Hara R, Simpson G, Solymos P, Stevens H, Wagner H 2012 Vegan: community ecology package. R package version 1 17-11.

[55] Eren AM, Maignien L, Sul WJ, Murphy LG, Grim SL, Morrison HG, Sogin ML 2013 Oligotyping: differentiating between closely related microbial taxa using 16S rRNA gene data. Methods Ecol Evol 4:1111–1119. doi:10.1111/2041-210X.12114. PMC3864673 24358444

