# Supplementary material for: Subtle Microbiome Manipulation Using Probiotics Reduces Antibiotic-Associated Mortality in Fish
Source: mSystems. 2017 Nov 7;2(6):e00133-17. doi: 10.1128/mSystems.00133-17 (PMC5675916; doi:10.1128/mSystems.00133-17)
Supplement: FIG S1 [file sys006172147sf1.pdf]

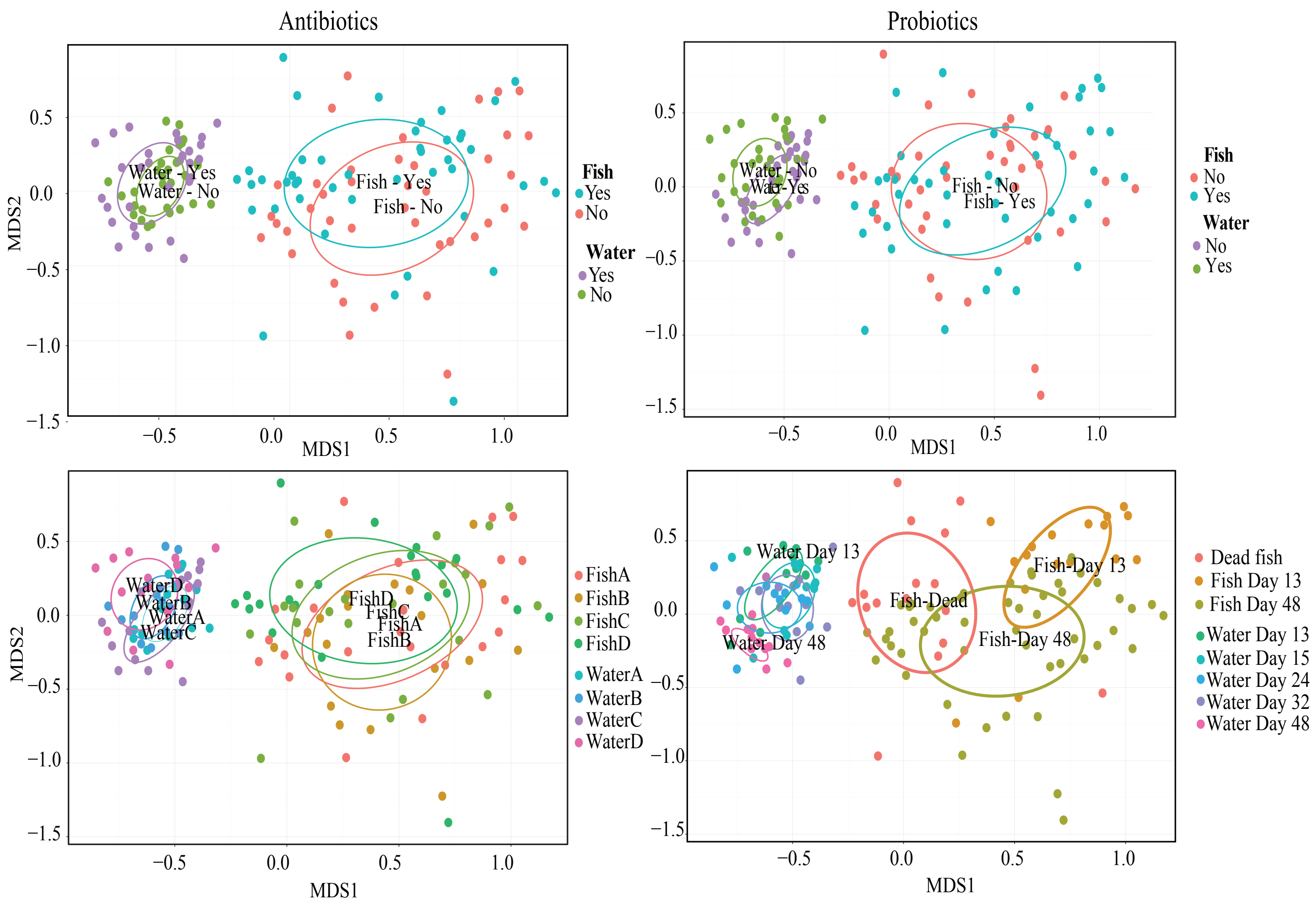

Figure S1: NMDS plots illustrating the influence of Antibiotics (A) Probiotics (B), Treatment (C) and collection date/mortality (D) on bacterial community structure across all collection days and samples. Note that only two dates are labeled for water collection date (D). 'Yes' and 'No' refer to the presence or absence of antibiotics (A) or probiotics (B).
