# Supplementary material for: Subtle Microbiome Manipulation Using Probiotics Reduces Antibiotic-Associated Mortality in Fish
Source: mSystems. 2017 Nov 7;2(6):e00133-17. doi: 10.1128/mSystems.00133-17 (PMC5675916; doi:10.1128/mSystems.00133-17)
Supplement: FIG S2 [file sys006172147sf2.pdf]

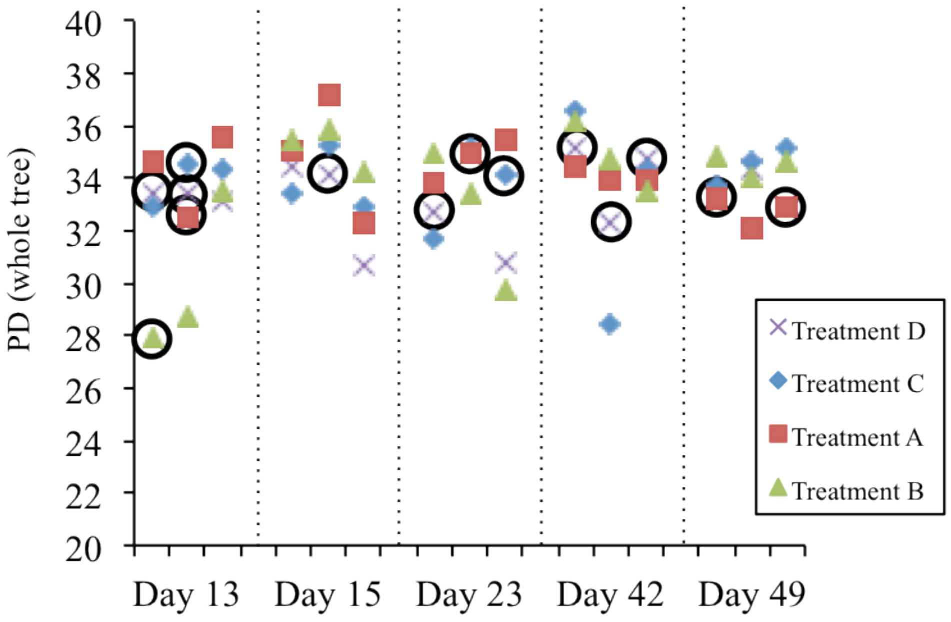

Figure S2: Richness of each water sample taken on a given day, colored by treatment and ordered by tank number (i.e. first column = tank 1, last = tank 3). Black circles indicate a mortality occurred near that sampling day.
