# Supplementary material for: Subtle Microbiome Manipulation Using Probiotics Reduces Antibiotic-Associated Mortality in Fish
Source: mSystems. 2017 Nov 7;2(6):e00133-17. doi: 10.1128/mSystems.00133-17 (PMC5675916; doi:10.1128/mSystems.00133-17)
Supplement: TEXT S1 [file sys006172147s5.docx]

Supplementary Material: Subtle microbiome manipulation using probiotics reduces antibiotic-associated mortality in fish

***Supplementary Methods***

*Genomic DNA extraction and 16S rRNA gene hypervariable region V6 amplicon library preparation*

We chose to homogenize the entire fish because the route of initial *Vibrio* colonization and infection is controversial, and by using only a single organ we may have missed the signal of infection. Homogenization of the entire carcass allows the fish microbiome to be sampled in aggregate, with no bias depending on what organ was chosen.

We homogenized entire fish carcasses after euthanization in 30 ml of sterile 1X Phosphate Buffered Saline (PBS) using dissection scissors. After homogenization we vortexed the resulting homogenate in a 50 ml sterile tube secured to a MoBio (Carlsbad, CA) Vortex-Genie2^®^ for 10 minutes to disassociate bacterial cells from host tissue and allowed the homogenate to settle. After < 60 seconds, we poured off 15 ml of homogenate (avoiding large chunks of fish tissue) and spun the resulting slurry at 16,000 x g for 10 minutes to pellet remaining fish tissue and all bacterial cells. Finally, we rinsed the pellet in 1X PBS, resuspended it in MoBio (Carlesbad, CA) PowerBiofilm Solution 1, and added the entire pellet to the bead tube. Extractions then proceeded as per manufacturer instructions.

To extract gDNA from water filtered through 0.2 μm Sterivex^TM^ filters (Millipore, Billerica, MA), we removed the filter paper from the cartridge and placed it in PureGene’s Yeast/Bac Kit Cell Lysis Solution (Qiagen, Hilden, Germany) with 0.1 mm MoBio DNA extraction beads. We vortexed tubes for 60 seconds to mechanically remove bacteria from the filter paper with the beads, then proceeded as per the PureGene extraction protocol.

***Supplementary Results***

*OTU level analyses – Fish microbiomes*

The top ten most abundant OTUs, as measured by mean relative abundance, accounted for nearly 60% of all reads from fish microbiome samples, and included six classes across three phyla: Clostridia, Bacilli (Firmicutes), Flavobacterium (Bacteroidetes), Betaproteobacteria, Alphaproteobacteria and Gammaproteobacteria (Proteobacteria), and Verrucomicrobia (Verrucomicrobia) (Figure 5).

Two Verrucomicrobia OTUs were the most abundant OTUs in our dataset (*Rubritalea* OTU 86 and *Rubritalea* OTU 963) and represented a combined mean relative abundance of 0.29 (SE +/- 0.013), reaching as high as 0.90 in a single sample (Figure S3). Although their combined relative abundance did not differ significantly between treatments they did display a significant inverse relationship related to tank membership (linear regression *P <* 0.01), meaning only one of the two OTUs is ever found at high abundance in a given tank (Figure S3).

Comparisons between the microbiome communities of fish treated with antibiotics (treatments C,D) and those not treated with antibiotics (A,B) found a surprising lack of variation. Of the 355 OTUs found in fish microbiomes after antibiotic and probiotic treatments (Day 13), none showed significantly different distributions in antibiotic versus non-antibiotic treated fish at an uncorrected alpha of 0.05. Of these 355 OTUs, only 12 were significantly different in fish microbiome communities exposed to probiotics (A,C) versus those without probiotics (B,D), and these included the probiotics themselves.

Comparisons between the microbiome communities of fish collected alive at the two sampling dates did reveal variation in several taxa. Flavobacteriaceae and Rhodeobacteracea were represented by 61 total OTUs (nearly 20% of all total OTUs), and both families showed significantly higher relative abundance in fish collected directly after antibiotic and probiotic treatments (Day 13) versus those collected at the end of the experiment (Day 48). We note this effect is not due to the probiotic or antibiotic administrations themselves, as no difference exists between treatments. However, both taxa also showed a reduction in relative abundance in fish that died during the course of the experiment (ANOVA *P <* 0.05).

Significant differences in the relative abundance of OTUs belonging to the family *Vibrionaceae* existed in comparisons between the microbiomes of fish collected alive, versus those collected dead. The average relative abundance of all Vibrionaceae in fish collected alive on Day 13 and Day 49 was 0.027 (SE 0.022) and 0.047 (SE 0.011) respectively, versus 0.274 (SE 0.052) in fish collected after mortality. This represents an order of magnitude increase in the relative abundance of Vibrionaceae OTUs in fish collected dead versus alive. We note that not all Vibrionaceae OTUs followed this general trend, but two in particular (OTUs 4696 and 4693) were significantly overrepresented in fish collected after mortality.

*OTU level analyses – Tank water microbiomes*

Water samples were taken at five time points in each tank. Bacterial communities were highly distinct from those found in fish, and no significant differences were found in regards to treatment or tank (Figure S4). Water bacterial communities were dominated by OTUs from three families, Rhodobacteraceae, Flavobacteriaceae, and Alteromonadaceae, representing a cumulative average relative abundance of nearly 50% (Figure S4). Interestingly, water communities were dynamic through time, with the most abundant OTU at a given time point often rare in others. This scenario is well illustrated by the two most abundant OTUs across all samples, Alteromonadaceae OTU 14 and Flavobacteriaceae OTU 648, which show considerable dynamism thru time across all tanks (Figure S4).

Physical and chemical parameters of the water were monitored throughout the study, and no significant differences were found between temperature and salinity that may explain variation of this kind through time. We note that ammonia and nitrate levels were measured using qualitative metrics, and we were only able to assure that levels stayed below 0.25 ppm and 5 ppm respectively in all tanks at all times points.

*Meta-analysis of probiotic OTUs*

In order to gain some insight into the ecology of our probiotic species beyond our experiment, we surveyed the NCBI nr database, and the VAMPS 16S rRNA gene amplicon database for exact matches to each OTU’s representative sequence. We found that the representative sequence for *Phaeobacter*sp. S4 was generally restricted to marine hosts and seawater. Of the 149 perfect matches to nr, 34% had been isolated from marine host environments, 29% from seawater, 11% form other marine sources (sediment, biofilm), while 26% had no source listed. The restriction of *Phaeobacter*sp. S4 to marine habitats was confirmed by a search of the VAMPS database, which showed the only projects with samples that had greater than 1% relative abundance of this OTU were marine water and marine plastic debris samples from projects LAZ_SEA and ICM_BMO (accessed via vamps.mbl.edu).

Conversely, the representative sequence from *B.* *pumilus* RI06-95 was remarkably cosmopolitan. Of the 1000 perfect matches we examined, only 1.8% came from marine host environments, 1.4% from seawater, and 3.1% from other marine sources. Over 80% came from terrestrial sources, including the surface of the Mars Exploration Rover (Acc # KT719510), soil, rhizosphere, mammal and insect guts, and fresh vegetables. Our VAMPS search of the same sequence revealed an equally cosmopolitan distribution, occurring in over 1,700 samples across 120 unique projects. Among the projects where the sequence occurred at greater than 1% relative abundance were the gut of birds, raw sewage, and the Phoenix spacecraft surface. We note it was not found above this abundance threshold in previous studies of *P. sphenops* microbiomes.
