# Supplementary material for: Subtle Microbiome Manipulation Using Probiotics Reduces Antibiotic-Associated Mortality in Fish
Source: mSystems. 2017 Nov 7;2(6):e00133-17. doi: 10.1128/mSystems.00133-17 (PMC5675916; doi:10.1128/mSystems.00133-17)
Supplement: FIG S3 [file sys006172147sf3.pdf]

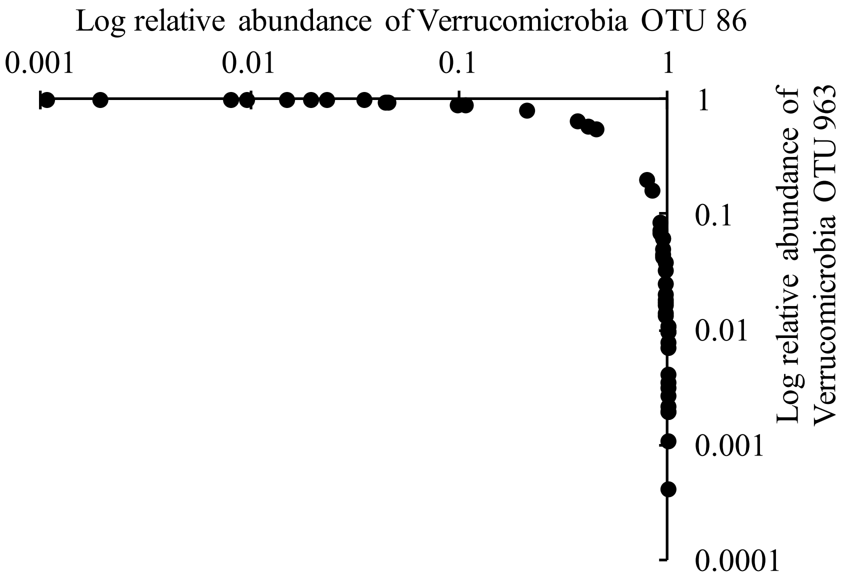

Figure S3: Relative abundance of the two most abundant OTUs across all fish samples. Each point is a single sample, with its relative abundance from each OTU plotted. These two OTUs show a strongly negative correlation to one another, a pattern driven by tank membership.
