# Supplementary material for: Subtle Microbiome Manipulation Using Probiotics Reduces Antibiotic-Associated Mortality in Fish
Source: mSystems. 2017 Nov 7;2(6):e00133-17. doi: 10.1128/mSystems.00133-17 (PMC5675916; doi:10.1128/mSystems.00133-17)
Supplement: FIG S4 [file sys006172147sf4.pdf]

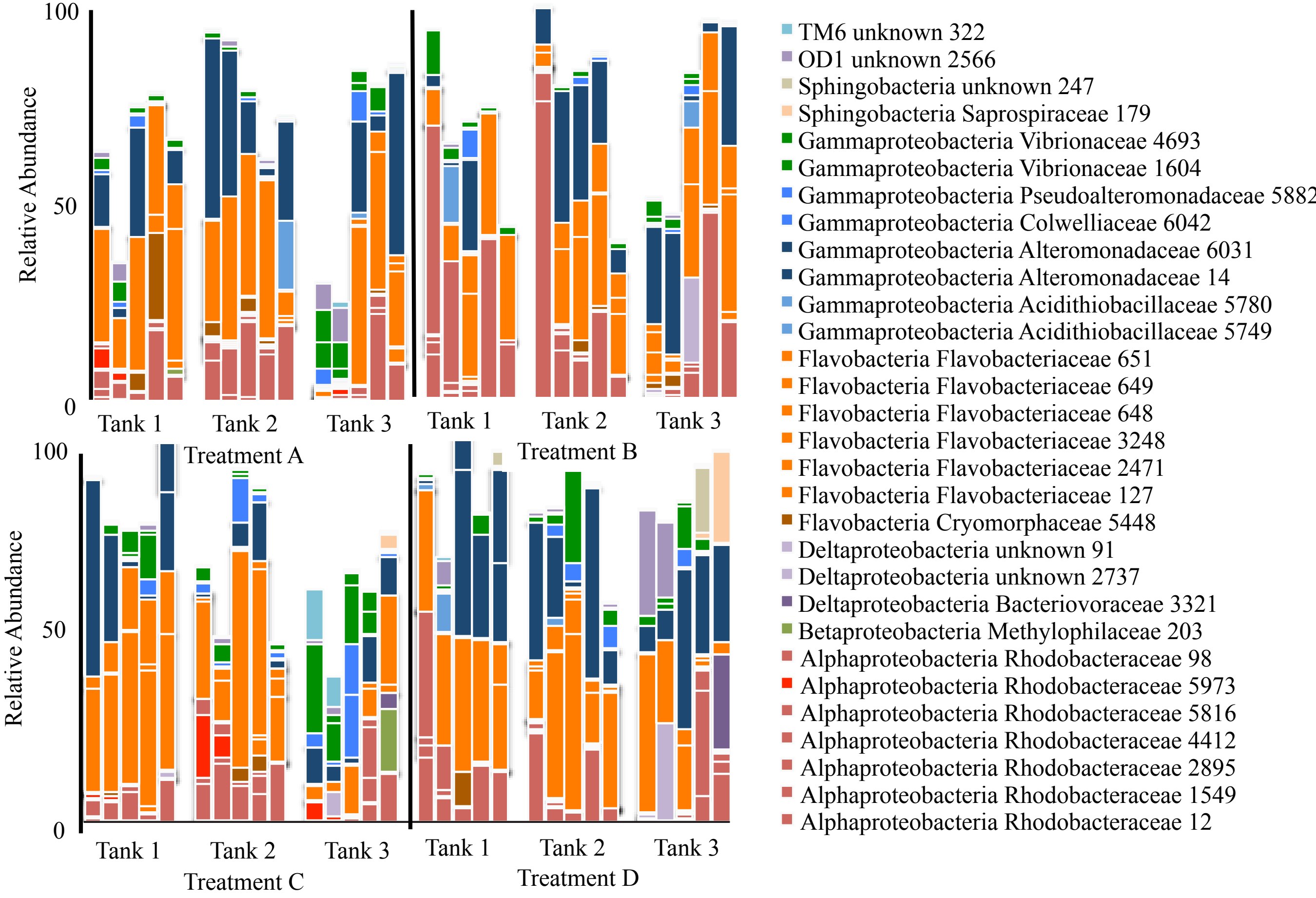

Figure S4: Relative abundance of the top 10 most abundant OTUs in each water sample across treatments and time. Each tank has 5 samples corresponding to the five sampling points (Days 12, 15, 24, 32, 48). More than 10 OTUs are shown since not all treatments had the same 10 top OTUs. OTUs are colored by family, with our *Phaeobacter* sp. S4 (OTU 5973) shown in bring red.
